# Postbiotic mediators derived from *Lactobacillus* species enhance riboflavin-mediated antimicrobial photodynamic therapy for eradication of *Streptococcus mutans* planktonic and biofilm growth

**DOI:** 10.1186/s12903-024-04620-z

**Published:** 2024-07-24

**Authors:** Maryam Pourhajibagher, Hassan-Ali Ghafari, Abbas Bahador

**Affiliations:** 1 Dental Research Center, Dentistry Research Institute, Tehran University of Medical Sciences, Tehran, Iran; 2 Department of Orthodontics, School of Dentistry, Shahed University, Tehran, Iran; 3 Department of Microbiology, School of Medicine, Tehran University of Medical Sciences, Tehran, Iran; 4 Fellowship in Clinical Laboratory Sciences, BioHealth Lab, Tehran, Iran

**Keywords:** Postbiotics, Antimicrobial photodynamic therapy, *Streptococcus mutans*, Biofilm, Dental caries

## Abstract

**Background:**

*Streptococcus mutans* has been implicated as a primary causative agent of dental caries and one of its important virulence properties is an ability to form biofilm on tooth surfaces. Thus, strategies to prevent and control *S. mutans* biofilms are requested. The present study aimed to examine the eradication of *S. mutans* planktonic and biofilm cells using riboflavin (Rib)-mediated antimicrobial photodynamic therapy (aPDT) enhanced by postbiotic mediators derived from *Lactobacillus* species.

**Materials and methods:**

Minimum inhibitory concentration (MIC) and the minimum bactericidal concentration (MBC) of Rib and postbiotic mediators were determined. The antimicrobial and anti-biofilm effects of Rib-mediated aPDT (Rib plus blue light), Rib-mediated aPDT in combination with postbiotic mediators derived from *Lactobacillus casei* (LC) (aPDT^+ LC^), and Rib-mediated aPDT in combination with postbiotic mediators derived from *Lactobacillus plantarum* (LP) (aPDT^+ LP^) were evaluated. The anti-virulence potential of Rib-mediated aPDT, aPDT^+ LC^, and aPDT^+ LP^ were assessed by measuring the expression of the *gtfB* gene using quantitative real-time polymerase chain reaction (qRT-PCR) at the highest concentrations of Rib, LC, and LP, at which the *S. mutans* had proliferation as the same as in the control (non-treated) group.

**Results:**

According to the results, the MIC doses of LC, LP, and Rib were 64 µg/mL, 128 µg/mL, and 128 µg/mL, respectively, while the MBC values of LC, LP, and Rib were 128 µg/mL, 256 µg/mL, and 256 µg/mL, respectively. Rib-mediated aPDT, aPDT^+ LP^, and aPDT^+ LC^ showed a significant reduction in Log_10_ CFU/mL of *S. mutans* compared to the control group (4.2, 4.9, and 5.2 Log_10_ CFU/mL, respectively; all *P* < 0.05). The most destruction of *S. mutans* biofilms was observed after treatment with aPDT^+ LC^ followed by aPDT^+ LP^ and Rib-mediated aPDT (77.5%, 73.3%, and 67.6%, respectively; all *P* < 0.05). The concentrations of 31.2 µg/mL, 62.5 µg/mL, and 62.5 µg/mL were considered as the highest concentrations of LC, LP, and Rib, respectively, at which *S. mutans* replicates as same as the control group and were used for *gtfB* gene expression assay using qRT-PCR during Rib-mediated aPDT, aPDT^+ LP^, and aPDT^+ LC^ treatments. Gene expression results revealed that aPDT^+ LP^ and aPDT^+ LC^ could decrease the gene expression level of *gtfB* by 6.3- and 5.7-fold, respectively (*P* < 0.05), while only 5.1-fold reduction was observed after Rib-mediated aPDT (*P* < 0.05).

**Conclusion:**

Our findings indicate that aPDT^+ LP^ and aPDT^+ LC^ hold promise for use as a treatment to combat *S. mutans* planktonic and biofilms growth as well as anti-virulence as a preventive strategy to inhibit biofilms development via reduction of *gtfB* gene expression.

## Introduction

Dental caries is a prevalent oral health issue worldwide. It is essentially a microbial biofilm-induced disease, that causes the demineralization of tooth enamel, leading to cavities and potentially severe consequences if left untreated [1]. Therefore, effective control of microbial biofilms is crucial to prevent dental caries. To achieve this, manual or electric toothbrushing is recommended as the primary method for both preventing and reducing microbial plaque accumulation [2]. While electric toothbrushes offer advantages like thorough cleaning and convenience, it’s essential to consider the limitations including higher cost, dependency on power, learning curve, maintenance requirements, and potential for excessive brushing [3]. However, this may not be feasible in patients who are physically or mentally disabled, as well as in post-surgical situations where oral hygiene is challenging. In these cases, alternative approaches to biofilm management may be necessary to prevent the development of dental caries. These approaches, though effective, have their own limitations that should be considered. Fluoride is a well-established and widely used preventive measure against tooth decay. It can be applied topically through toothpaste, mouthwash, or professional treatments. Fluoride helps to inhibit tooth demineralization, promote tooth remineralization, and inhibit plaque bacteria. Overexposure to fluoride can lead to fluorosis, a condition characterized by white or brown spots on the teeth. Fluoride is less effective in individuals with high-sugar diets or poor oral hygiene [4, 5]. Dental sealant application is another prevention strategy against dental caries. They are applied to the chewing surfaces of teeth, particularly molars and premolars. They act as a physical barrier, preventing bacteria and food particles from accumulating in the grooves and crevices. Sealants are not effective for teeth with existing cavities. They may need to be reapplied over time as they can wear off [6]. Xylitol, a sugar substitute, has been shown to inhibit the growth of bacteria that contribute to tooth decay. High consumption of xylitol can cause gastrointestinal issues including diarrhea and stomach disturbance. Its effectiveness may vary depending on individual oral health [7]. Antibacterial agents, such as chlorhexidine, can be used to reduce bacterial populations in the mouth. Some of the most frequent adverse effects of chlorhexidine (CHX) mouthwash include taste alteration, xerostomia, numbness in the mouth and tongue, hypogeusia, calculus, and extrinsic tooth staining [8]. Probiotics, beneficial bacteria, can help maintain a healthy oral microbiome, reducing the risk of dental caries. More research is needed to fully understand their effectiveness. Probiotics may not be effective for individuals with severe oral health issues. Due to the challenges of maintaining the viability of probiotics in food until consumption and under unfavorable environmental and gastrointestinal conditions, there is growing interest in utilizing probiotic byproducts and secretions, particularly postbiotics [9]. Based on the limitations of the strategies mentioned, it is crucial to develop an effective approach to prevent and manage dental caries.

*Streptococcus mutans* plays a significant role in the development of dental caries by initiating the formation of microbial biofilms, which are a primary trigger for the progression of dental caries. *S. mutans* can produce insoluble extracellular polysaccharides by metabolizing dietary sucrose [10]. This can increase the ability of bacteria to attach to teeth and form biofilms. The stronger adherence is mediated by glucosyltransferases (Gtfs), especially GtfB [11]. *gtfB* gene encodes glucosyltransferases 1, which primarily synthesizes mostly insoluble glucans containing α-1,3-linked glucose. It facilitates bacterial adherence and accumulation on tooth surfaces, allowing vertical growth of the microcolonies, increasing the thickness of the cariogenic biofilms, and resulting in dental caries. The GtfB protein’s ability to attach to the surface of *Actinomyces* spp. and oral streptococci increases the number of insoluble glucans produced, which promotes the binding of *S. mutans* to other organisms and provides additional structural support for microcolony development. This effect could explain the formation of highly organized, large microcolonies surrounded by an intricate web-like exopolysaccharide matrix observed in the late stages of biofilm formation. Therefore, the *gtfB* gene has become a potential target for protection against dental caries [12].

Antimicrobial photodynamic therapy (aPDT) is a promising approach to eradicate both microbial planktonic and biofilm cells [13]. Microbial biofilms are communities of microorganisms that are highly resistant to traditional antimicrobial treatments. Compared to planktonic cells, bacterial cells within biofilms are much better protected from attack by noxious agents [14]. aPDT attacks many components of the biofilm, including proteins, lipids, and nucleic acids present within the biofilm matrix, causing damage to the biofilm structure [15]. In aPDT, uses a photosensitizer and an appropriate wavelength of light source in the oxygen microenvironment to generate reactive oxygen species (ROS) that can inactivate bacteria [16]. The ROS can be a novel solution to overcome the factors that increase the resistance of biofilms to antimicrobial treatments. aPDT photochemistry reactions to promote microbial cell damage are defined as type I and II mechanisms. The type-I photochemical reaction is the dominant process, where the intracellular localization of the photosensitizer greatly determines the site of cellular damage. Additionally, a type-II photochemical pathway has been proposed, which involves oxygen-independent photoinactivation of microorganisms through photoinduced electron transfer that produces reactive inorganic radicals. The short lifetime and diffusion distance of ROS hinder their reach to pathogens localized far from the site of ROS production [15].

Recently, natural photosensitizers have been studied for use in aPDT, and research has shown that they can be effective in killing microorganisms [17]. Riboflavin (Rib), more commonly known as vitamin B2, is a natural and effective photosensitizer. The Rib has two absorbance peaks at 360 and 440 nm [18]. Rib is capable of producing ROS under blue light exposure thereby inducing oxidative damage in tissues resulting in cellular damage [19]. It has been studied extensively for its potential use in aPDT against oral infections [20], and the results have been promising. aPDT can be combined with other antimicrobials including probiotics [21, 22], antibiotics [23, 24], chitosan [25], β-cyclodextrin [26], and potassium iodide [27] to enhance its efficacy and modulate the oral microbiome and promote a healthy balance of bacteria [28].

According to the International Scientific Association for Probiotics and Prebiotics (ISAPP) the term “postbiotics”, also known as postbiotics mediators, refers to inanimate microorganisms, the metabolic and secretory products produced by microbes during growth and fermentation that remain after a live microbe have been subjected to an inactivation process. These products include cell wall fragments, short-chain fatty acids (SCFAs), exopolysaccharides (EPS), vitamins, bacteriocins, enzymes, and peptides [29, 30]. Postbiotics are distinct from probiotics and prebiotics, as they do not involve live microorganisms or non-digestible food ingredients, but rather focus on the health benefits derived from inanimate microorganisms or their components [31–34]. Postbiotics have gained considerable attention in recent years for their potential health benefits, including their role in the prevention of dental caries [9, 35]. They have been found to suppress the growth and activity of cariogenic bacteria, such as *S. mutans*, which is a major contributor to dental caries [35]. This can be achieved through the production of antimicrobial peptides, organic acids, and hydrogen peroxide. These substances inhibit the growth and colonization of cariogenic bacteria, reducing the risk of dental caries. Incorporating postbiotics into oral health strategies may provide a new avenue for preventing dental caries, but further studies are needed to validate their efficacy and explore their practical applications [35, 36].

According to the literature, there have been no studies investigating the combined antimicrobial effect of aPDT and postbiotics against oral pathogens. Therefore, this study aims to examine the eradication of *S. mutans* planktonic and biofilm growth using Rib-mediated aPDT enhanced by postbiotic mediators derived from *Lactobacillus casei* and *L. plantarum*.

## Materials and methods

### Preparation of postbiotic mediators derived from *Lactobacillus casei* (LC) and *Lactobacillus plantarum* subsp. *Plantarum* (LP)

To prepare postbiotics derived from *Lactobacillus casei* (LC) ATCC 393 and *Lactobacillus plantarum* subsp. *Plantarum* (LP) ATCC 14,917 (obtained from Iranian Biological Resource Center [IBRC]), a single colony of LC and LP separately grown in de Man, Rogosa, and Sharpe (MRS) broth (Merck, Darmstadt, Germany) at 37 °C for 36 h in the presence of 5% CO_2_. After that, the cell-free culture supernatants of LC and LP were obtained by separating them from the bacterial cells through centrifugation at 10,000×g for 10 min at 4 °C. The supernatants were filtered using a 0.2 μm membrane filter to remove any remaining bacterial cells. The filtered supernatants were then freeze-dried and were stored at -20 °C. Before use, these powders were rehydrated through the addition of sterile deionized water.

### Bacterial strains and growth conditions

*S. mutans* ATCC 35,668, purchased from IBRC, was cultured on brain heart infusion (BHI) agar (Merck, Darmstadt, Germany) at 37 °C for 24 h with 5% CO_2_. A colony of *S. mutans* was then picked up from the BHI agar and transferred into a tube containing BHI broth (Merck, Darmstadt, Germany). The tube was incubated in a shaker incubator at 120 rpm and 37 °C for 24 h until the bacterial population reached half of the McFarland standard, equivalent to 1.5 × 10^8^ colony forming units per milliliter (CFU/mL).

### Photosensitizer and light source

The Rib (Sigma, Germany) compound was utilized as a photosensitizing agent. A stock solution of Rib (2 mg/mL) was made in sterile deionized water and stored in the dark at 4 °C before use. This concentration of riboflavin was based on its maximum solubility in deionized water at 100 °C [37]. The stock solution was passed through a 0.2 μm membrane filter for sterilization and then further diluted in sterile deionized water to achieve the different test concentrations.

A light-emitting diode (LED, DY400–4, Denjoy, China) at the wavelength of 450 ± 30 nm with an output intensity of 1.0 ± 1.4 W/cm^2^, an energy density of 60–80 J/cm^2^, and an exposure time of one minute was used as the light source.

### Minimum inhibitory concentration (MIC) and the minimum bactericidal concentration (MBC) of Rib and postbiotic mediators

The MIC values of Rib and postbiotic mediators derived from LC and LP against *S. mutans* strain were determined by the Mueller-Hinton (MH) broth microdilution method, which follows the guidelines of the Clinical and Laboratory Standards Institute (CLSI) [38]. Serial dilutions were made in MH broth medium (Merck, Darmstadt, Germany) in a 96-well microtiter plate. The dilution ranges of Rib and postbiotic mediators was 1–512 µg/mL. After that, 100 µL of *S. mutans* suspension with a final concentration of 1.5 × 10^6^ CFU/mL was added to each dilution. The microtiter plate was then incubated for 24 h at 37 °C in the presence of 5% CO_2_, and the MIC was defined as the concentration of Rib and postbiotic mediators that will inhibit the visible growth of a microorganism. The well containing a bacterial suspension and MH broth without Rib and postbiotic mediators was considered the positive control, while the negative control was the well-containing Rib and postbiotic mediators and MH broth without a bacterial suspension. MBC values of Rib and postbiotic mediators were determined by re-culturing broth dilutions that inhibit bacterial growth, typically those at or above the MICs. The MBC is defined as the lowest concentration of the agents that reduces the viability of the initial bacterial inoculum by at least 99.9%.

### Determination of viability of *S. mutans* following treatment with different concentrations of Rib and postbiotic mediators

90 µL of Rib, LC, and LP (at the concentrations of 2000 µg/mL) were added to the well in column one of a 96-well microtiter plate, separately, and were diluted two-fold stepwise after adding 90 µL of BHI broth to each well. Then, 10 µL of 1.5 × 10^6^ CFU/mL of *S. mutans* suspension was poured into each well. Then microtiter plate was incubated for 24 h at 37 °C in the presence of 5% CO_2_. Thereafter, 10 µL of each well was cultured in a BHI agar plate using the spread technique with an L-shaped bar. The BHI agar plates were incubated for 24 h at 37 °C in the presence of 5% CO_2_, and the CFU/mL was determined using the previous study [39].

### Study design

#### Effects of different treatment groups on the planktonic growth of *S. mutans*

The effects of the treatments on the planktonic growth of *S. mutans* were determined as described previously [40]. In brief, 10 µL of *S. mutans* suspension at a concentration of 1.5 × 10^6^ CFU/mL was added to the wells of a 96-well microtiter plate. The bacterial cells were then treated in the experimental groups as described below:

A. **Rib**: 100 µL of Rib at 1/2×MIC (64 µg/mL) was added to the bacterial cells and the microtiter plate was incubated in the dark at room temperature for 5 min.
B. **LC**: 100 µL of LC at 1/2×MIC (32 µg/mL) was added to the bacterial cells and the microtiter plate was incubated in the dark at room temperature for 5 min.
C. **LP**: 100 µL of LP at 1/2×MIC (64 µg/mL) was added to the bacterial cells and the microtiter plate was incubated for 5 min.
D. **Blue light**: 100 µL of BHI broth was added to the bacterial cells and *S. mutans* was exposed to the blue light at the wavelength of 450 ± 30 nm for one minute. Black paper was used under a microtiter plate to prevent beam reflection and neighboring wells were filled with methylene blue to prevent transmission of light. The probe of the laser was fixed 2 mm above the top surface of the microplate by a stand [39].
E. **aPDT**: *S. mutans* suspension was treated by Rib similar to Group A, and before light irradiation similar to Group D, excess Rib in the growth medium of bacteria was removed by gently washing 3 times with 300 µL of sterile phosphate-buffered saline (PBS; pH: 7 ± 0.5).
F. **aPDT**^**+ LC**^**and aPDT**^**+ LP**^**(aPDT in combination with postbiotic mediators derived from LP and LC)**: *S. mutans* suspension was treated by aPDT similar to Group E, and the cells were separately exposed to LC and LP similar to Groups B and C, respectively.
G. **Positive control**: 100 µL of 0.2% CHX was added to the bacterial cells and incubated at room temperature for 5 min.
H. **Negative control**: 100 µL of normal saline was added to the bacterial cells and incubated for 5 min at room temperature.
I. After each treatment, the bacterial cells were serially 10-fold diluted with BHI broth. The diluted samples were then incubated at 37 °C for 24 h in the presence of 5% CO_2_, and the log_10_ CFU/mL values were calculated.

#### Effects of different treatment groups on the biofilm growth of *S. mutans*

To form biofilms, a previously described method was used to create a biofilm on the bottom of a 96-well flat-bottom microtiter plate [41]. Briefly, 200 µL of overnight culture of *S. mutans* was added to each well of the microtiter plate. The plate was incubated for 24 h at 37 °C for biofilm growth. After biofilm formation, the medium in each well was removed, and the wells were washed twice with PBS to remove any planktonic cells. The adherent bacteria in the biofilms were then treated with the experimental groups labeled as A-H, as mentioned above. The treated bacteria were fixed with 95% ethanol and stained with 0.1% crystal violet for 10 and 15 min, respectively. To measure biofilm degradation, the crystal violet dye was solubilized by adding 100 µL of acetic acid (33%). Finally, the absorbance of the solubilized dye was measured at 570 nm.

### Effect of treatments on the expression of the S. *mutans gtfB* gene

#### Determination of the highest concentrations of Rib, LC, and LP, at which the *S. mutans* had proliferation as the same as in the control (non-treated) group

Since, in the study of gene expression, any change in the microbial population as a confounding factor can affect the expression level of genes, in the current study, the anti-virulence effects of Rib-mediated aPDT, aPDT^+ LC^, and aPDT^+ LP^ were evaluated by measuring the expression of the *gtfB* gene using quantitative real-time polymerase chain reaction (qRT-PCR) at the highest concentrations of Rib, LC, and LP, at which the *S. mutans* had proliferation as the same as in the control (non-treated) group. This concentration of Rib was determined using a broth microdilution testing method described in a previous study [42]. Briefly, Rib (2 mg/mL) was diluted in two-fold serial dilutions with BHI broth in a 96-well microtiter plate. *S. mutans* suspension, with a concentration of 1.5 × 10^6^ CFU/mL, was then added to each well at a volume of 10 µL per well. To measure the reduction in the microbial count, after 24 h, 10 µL from each well with varying dilution series were spread onto BHI agar, and the number of CFU/mL was determined using the method of Miles et al. [43]. The highest concentrations of Rib, LC, and LP, at which the *S. mutans* had proliferation as the same as in the control group were determined like the measurement for Rib, except that LC and LP were used instead of the Rib.

#### RNA extraction and qRT-PCR

The gene expression involved in *S. mutans* biofilm formation (*gtfB*) was measured using qRT-PCR. After treating *S. mutans* with the experimental groups mentioned above, total RNA was extracted using the super RNA extraction kit (AnaCell, Iran) following the manufacturer’s instructions. The extracted RNA was then used to synthesize cDNA using the cDNA synthesis kit (AnaCell, Iran) as per the manufacturer’s recommendations. Specific primers related to the *gtfB* and *16 S rRNA* genes were designed using Primer3Plus software version 4.0 (Table 1). The real-time PCR reaction was conducted in the ABI Thermocycler System (ABI Step One™, USA) with the following cycle profile: One cycle at 95 °C for 5 min followed by 40 cycles at 95 °C for 20 s, annealing at 60 °C for 10 s, and extension at 72 °C for 10 s. The *16 S rRNA* expression was used to normalize and calculate the relative changes in target gene expression using the 2^−ΔΔCT^ method [44]. Control reactions were performed to ensure that no genomic DNA was amplified during the PCR process.

**Table 1:** Primer sequence for qRT-PCR analysis

Gene		Primer sequence (5′–3′)	Product size (bp)*
***gtfB***	Forward	TGTTGTTACTGCTAATGAAGAA	103
	Reverse	GCTACTGATTGTCGTTACTG	
***16SrRNA***	Forward	CCTACGGGAGGCAGCAGTAG	121
	Reverse	CAACAGAGCTTTACGATCCGAAA	

*bp: base pair

### Statistical analysis

All experiments were conducted in triplicate, and the data were denoted as mean ± standard deviation (SD). SPSS package program version 25.0 (IBM-SPSS, IBM Corp. Released, 2012) was applied to perform statistical analysis and the data were analyzed using one-way ANOVA with post-hoc comparisons. Results with a P value less than 0.05 were considered significant.

## Results

### MIC and MBC doses of Rib and postbiotic mediators

**Fig. 1:**
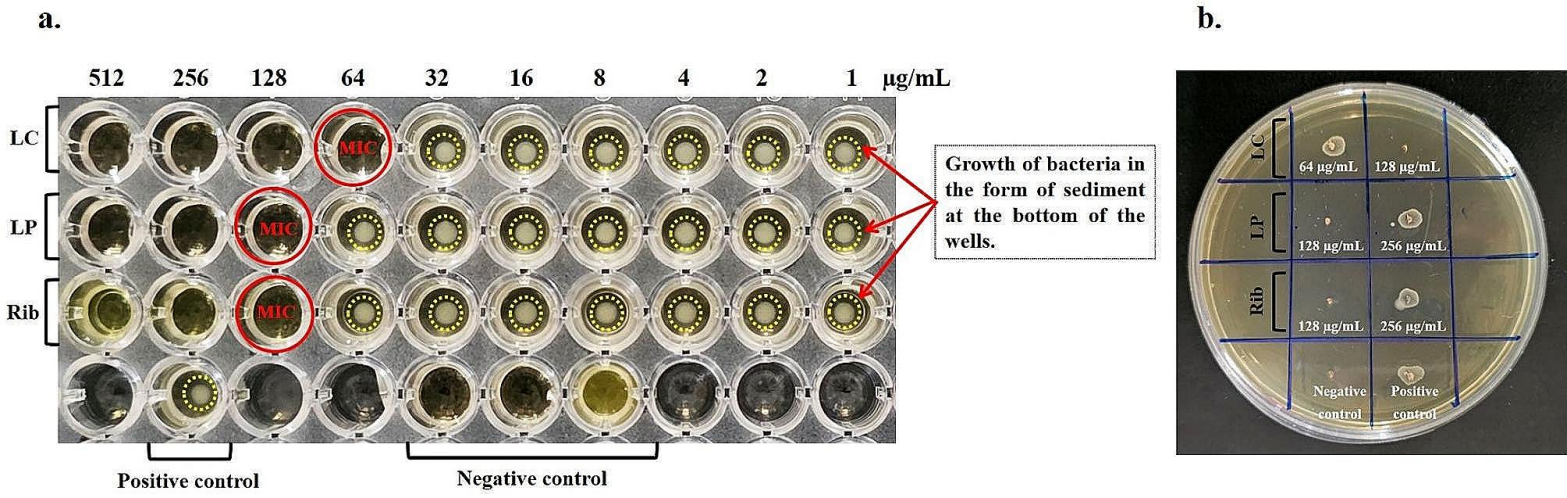
Determination of minimum inhibitory concentration (MIC) and minimum bactericidal concentration (MBC) of Riboflavin and postbiotic mediators against *Streptococcus mutans*. LC: *Lactobacillus casei*, LP: *Lactobacillus plantarum* subsp. *Plantarum*, Rib: Riboflavin

In this study, the antibacterial activity of different concentrations of Rib, LC, and LP was tested against *S. mutans*. The growth turbidity in the broth was observed to evaluate the antibacterial activity. The results showed that the lowest LC, LP, and Rib concentrations that inhibited the visible growth (MIC) of *S. mutans* were 64 µg/mL, 128 µg/mL, and 128 µg/mL, respectively (Fig. 1a). Moreover, the MBC values of LC, LP, and Rib were 128 µg/mL, 256 µg/mL, and 256 µg/mL, respectively (Fig. 1b).

### Viability of *S. mutans* following treatment with different concentrations of Rib and postbiotic mediators

**Fig. 2:**
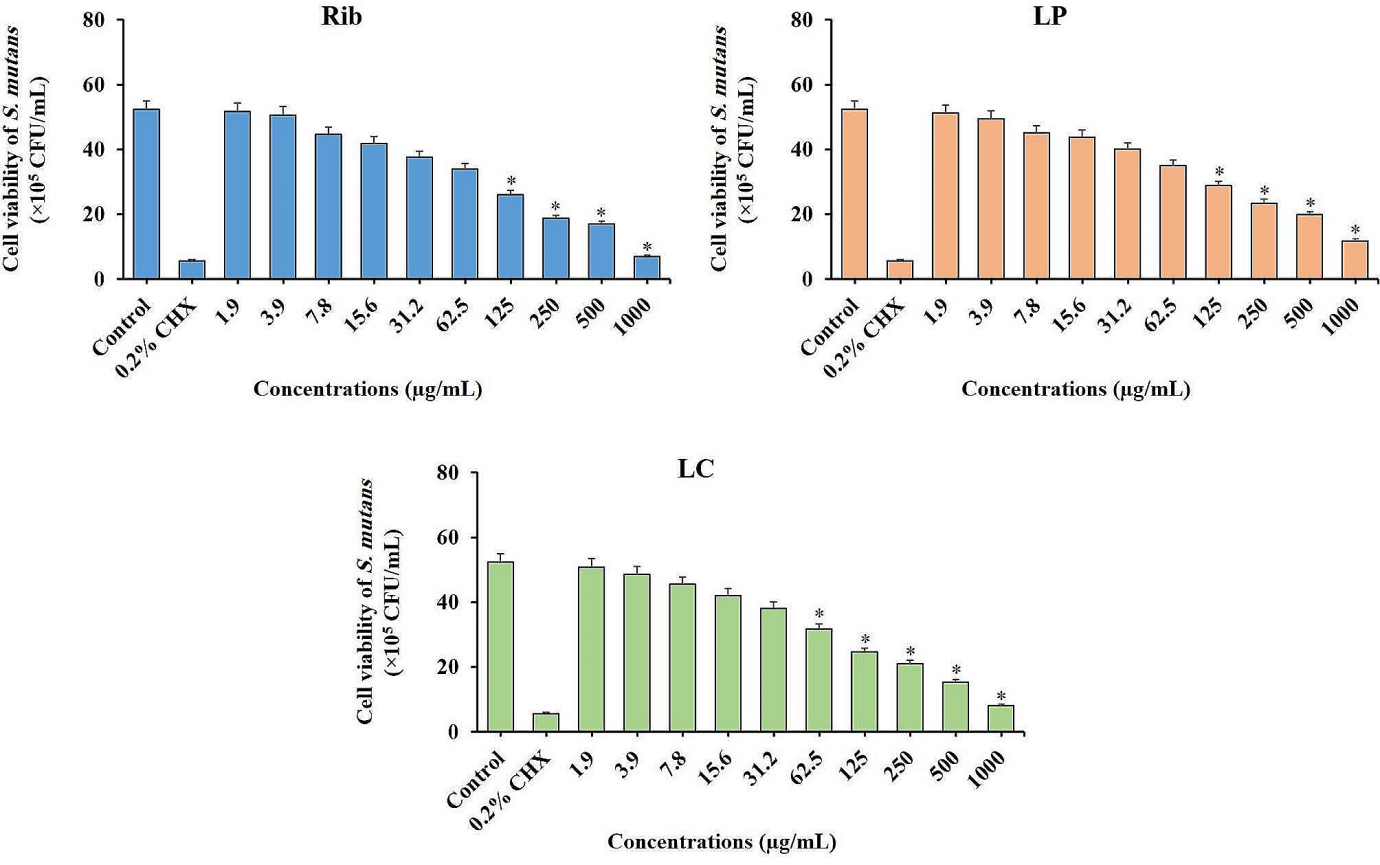
Effects of different concentrations of riboflavin (Rib), postbiotic mediators derived from *Lactobacillus plantarum* subsp. *Plantarum* (LP), and postbiotic mediators derived from *Lactobacillus casei* (LC) alone [all 1.9 to 1000 µg/mL] on cell viability of *Streptococcus mutans.* The concentrations higher than 62.5, 125, and 125 µg/mL were effective for LC, LP, and Rib, respectively, in significantly reducing the growth of *S. mutans*. *Significant different at *P* < 0.05

According to the results presented in Fig. 2, different concentrations of Rib, LC, and LP were examined to evaluate their effect on the bacterial growth of *S. mutans*. As the concentration of Rib increased, there was a reduction in the cell viability of *S. mutans* compared to the control group. The results showed that concentrations of Rib and LP between 125 and 1000 µg/mL were able to significantly reduce the bacterial growth of *S. mutans* (*P* < 0.05). However, concentrations of Rib and LP lower than 125 µg/mL (1.9–62.5 µg/mL) had no significant effect in reducing the viability of microbial cells (*P* > 0.05). Based on these results presented in Fig. 2, the concentration of 62.5 µg/mL was considered the highest concentration of Rib and LP, at which the *S. mutans* had proliferation as the same as in the control (non-treated) group. However, concentrations lower than 62.5 µg/mL (1.9–31.2 µg/mL) of LC had no significant effect in reducing the viability of *S. mutans* (*P* > 0.05). Based on these results, the concentrations of 31.2 µg/mL, 62.5 µg/mL, and 62.5 µg/mL were considered the highest concentrations of LC, LP, and Rib, respectively, at which the *S. mutans* had proliferation as the same as in the control group (Fig. 4).

**Fig. 3:**
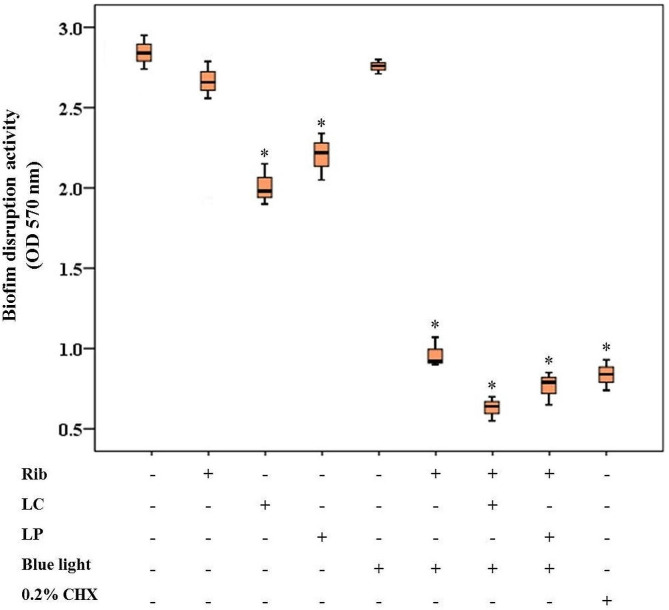
Effects of different treatment groups on cell viability of *Streptococcus mutans.* A statistically significant decrease was observed after the treatment with 0.2% chlorhexidine (CHX), aPDT (riboflavin [Rib] plus blue light irradiation) and aPDT^+ LP^ and aPDT^+ LC^ groups (aPDT in combination with postbiotic mediators derived from *Lactobacillus plantarum* subsp. *Plantarum* [LP] and aPDT in combination with postbiotic mediators derived from *Lactobacillus casei* [LC]), LP, and LC alone (*P* < 0.05). In the box plot, the upper and lower sides of the box represent the upper and lower quartiles, respectively. The box covers the interquartile interval, which contains 50% of the data. The median is represented by a horizontal bold black line that splits the box in two. The whiskers are the two lines outside the box, which extend from the minimum to the lower quartile (the start of the box) and from the upper quartile (the end of the box) to the maximum. *Significant different at *P* < 0.05

### Antibacterial effects of treatment groups figd planktonic growth of *S. mutans*

Our results in Fig. 3 demonstrated that there were significant reductions in cell viability of *S. mutans* following Rib-mediated aPDT, aPDT^+ LP^, and aPDT^+ LC^ groups versus the control group (*P* < 0.05). Figure 3 also shows that photokilling by aPDT was enhanced by the presence of the postbiotic mediators, although the postbiotic mediators alone had a nearly 2 Log_10_ CFU/mL killing effect (2.41 and 2.19 Log_10_ CFU/mL for LC and LP, respectively). It revealed that the effects of adding the postbiotic mediators were only additive. The study found that aPDT plus LP and LC could reduce the survival of *S. mutans* by 4.9 Log_10_ CFU/mL and 5.2 Log_10_ CFU/mL, respectively, while a 4.2 Log_10_ CFU/mL reduction was observed in aPDT using Rib group. Cell viability reduction was also observed in Rib, postbiotic mediators, and blue light alone, however, this difference was not significant (*P* > 0.05).

**Fig. 4:**
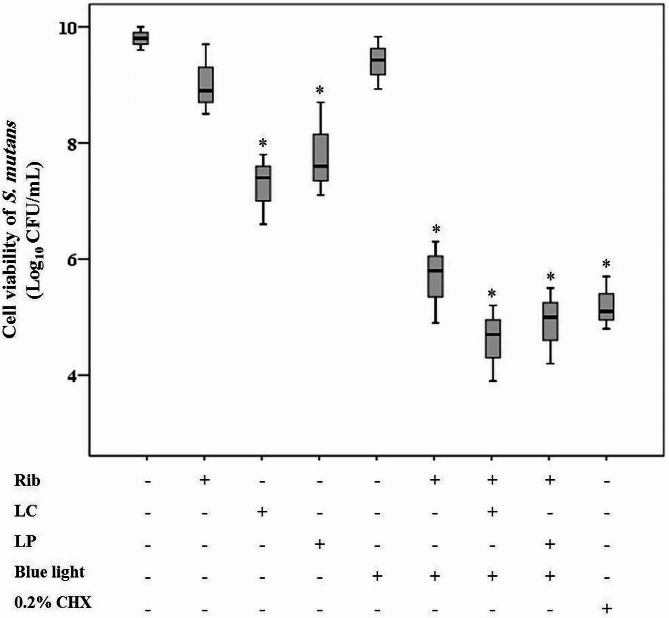
Effects of different treatment groups on biofilm disruption of *Streptococcus mutans* using crystal violet staining method. A statistically significant decrease was observed after the treatment with 0.2% chlorhexidine (CHX), aPDT (riboflavin [Rib] plus blue light irradiation) and aPDT^+ LP^ and aPDT^+ LC^ groups (aPDT in combination with postbiotic mediators derived from *Lactobacillus plantarum* subsp. *Plantarum* [LP] and aPDT in combination with postbiotic mediators derived from *Lactobacillus casei* [LC]), LP, and LC alone (*P* < 0.05). In the box plot, the upper and lower sides of the box represent the upper and lower quartiles, respectively. The box covers the interquartile interval, which contains 50% of the data. The median is represented by a horizontal bold black line that splits the box in two. The whiskers are the two lines outside the box, which extend from the minimum to the lower quartile (the start of the box) and from the upper quartile (the end of the box) to the maximum. *Significant different at *P* < 0.05

### Biofilm disruption effects of treatment groups

The crystal violet results in Fig. 4 showed that there was significant degradation of preformed biofilms in all treatment groups compared to the control group (*P* < 0.05), except for blue light (*P* > 0.05). In Fig. 4, the LC and LP disrupted the *S. mutans* biofilms by 30.28% and 21.83%, respectively, compared to the control group (*P* < 0.05). The study found that the Rib plus blue light irradiation (i.e., aPDT) had significant anti-biofilm effects against *S. mutans* (67.60% biofilms reduction) in comparison with the control group (*P* < 0.05). Additionally, when these mediators were combined with Rib mediated-aPDT, an additive effect was apparent. The highest amount of biofilm disruption was observed in aPDT^+ LC^ and aPDT^+ LP^ groups. aPDT^+ LC^ and aPDT^+ LP^ could destroy 77.5% and 73.3% of *S. mutans* biofilms, respectively, compared to the control group (*P* < 0.05). Although the biofilm reduction activity of CHX against *S. mutans* biofilms (70.8%) was higher and lower than Rib-mediated aPDT, aPDT^+ LP^, and aPDT^+ LC^ groups, respectively, these changes were not statistically significant (*P* > 0.05).

### Effect of treatments on the expression of the S. *mutans gtfB* gene

**Fig. 5:**
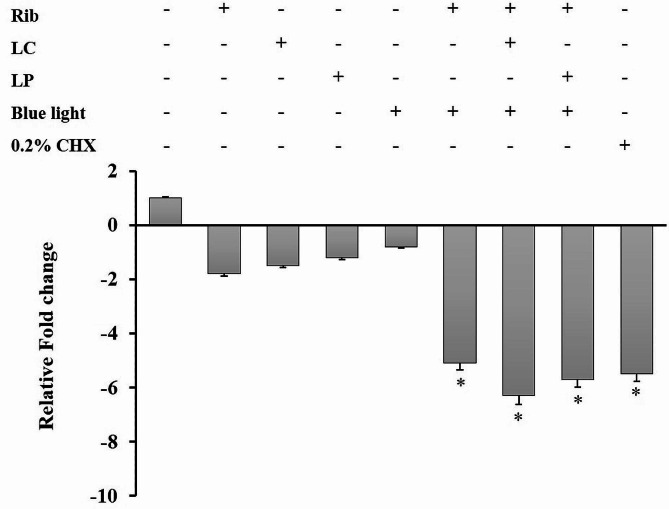
Effects of different treatment groups on the mRNA expression levels of *gtfB* gene in *Streptococcus mutans*. A statistically significant decrease was observed after the treatment with Rib-mediated aPDT (riboflavin [Rib] plus blue light irradiation), aPDT^+ LP^ (aPDT in combination with postbiotic mediators derived from *Lactobacillus plantarum* subsp. *Plantarum* [LP]), and aPDT^+ LC^ (aPDT in combination with postbiotic mediators derived from *Lactobacillus casei* [LC]), Rib, LP, and LC alone (*P* < 0.05). *Significant different at *P* < 0.05

The results in Fig. 5 showed that Rib, LC, and LP alone in the absence of light affected the expression of the *gtfB* mRNA gene in *S. mutans* compared to the control group (1.81-, 1.54-, and 1.32-fold reduction, respectively). The blue light alone also revealed a 0.81-fold reduction (*P* > 0.05) in *gtfB* mRNA gene expression in *S. mutans*, perhaps from interactions with intrinsic photosensitizers. The data showed that aPDT plus LC and aPDT plus LP could decrease the gene expression level of *gtfB* by 6.3- and 5.7-fold, respectively (*P* < 0.05), while only 5.1-fold reduction was observed after Rib-mediated aPDT (*P* < 0.05). The combined impact of LP and LC on the aPDT protocol had additive effects on the reduction of the expression of the *gtfB* mRNA gene. It is noteworthy that there is no significant difference in the reduction of gene expression between Rib-mediated aPDT, aPDT^+ LP^, and aPDT^+ LC^ groups with 0.2% CHX (*P* > 0.05).

## Discussion

The primary approaches for preventing dental caries include several strategies such as fluoride application, pit and fissure sealants, oral hygiene, a non-cariogenic diet and limiting sugar consumption, regular dental visits, and patient education. These preventive strategies are crucial for maintaining good oral health and reducing the incidence of dental caries. They should be integrated into individual and community-based policies to ensure comprehensive prevention and management of dental caries. In developing countries like Iran, the challenge of dental caries prevention is magnified by barriers such as low socioeconomic status (SES). Low SES has been identified as a significant predictor of oral health disparities, with individuals from lower-income households facing increased barriers to accessing dental care and preventive services. These barriers can include financial constraints, limited dental insurance coverage, transportation issues, language barriers, and a lack of awareness about the importance of oral health. Consequently, several studies have focused on approaches that may potentiate the activity of available low-cost photosensitizer in aPDT, in combination with other antimicrobials including probiotics [21, 22], antibiotics [23, 24], chitosan [25], β-cyclodextrin [26], and potassium iodide [27] to enhance its efficacy and modulate the oral microbiome and promote a healthy balance of bacteria [28]. The recent report of Global Oral Health Status Report (GOHSR) which was published by the World Health Organization in November 2022 has declared an urgent need for low-cost and highly effective strategies for improving dental health and prevention of dental caries for countries with lower gross domestic products, especially from a financial and logistics aspect [45]. Thus, to address the need for affordable and effective strategies to control dental caries, we investigated the combined antimicrobial effect of Rib-mediated aPDT and postbiotic mediators derived from *L. casei* and *L. plantarum* against the *S. mutans* planktonic and biofilm growth to explore a feasible adjunctive strategy for control dental caries in developing countries with limited resources.

Generally, studies on the antimicrobial effects of Rib are scarce. Our findings are consistent with a recent study that revealed Rib has an anti-bacterial effect [46]. It has been shown that Rib has broad-spectrum antimicrobial properties against pathogenic microbes including *Staphylococcus aureus*, *Enterococcus faecalis*, *Salmonella typhi*, *Klebsiella pneumonia*, *Pseudomonas aeruginosa*, *Candida albicans*, and *Plasmodium falciparum* [46]. In line with these findings, the results of the present study showed that Rib alone can inhibit the proliferation of *S. mutans*.

Recently, natural photosensitizers such as Rib, which is recognized by the FDA as generally safe, have gained attention due to their unique structural, antimicrobial, anti-cancer, and anti-inflammatory properties [18, 47–49]. The amount of microbial destruction via Rib-mediated aPDT around fixed orthodontic devices was assessed by the Kamran et al. study [19]. It was observed that aPDT using Rib and blue LED irradiation efficiently reduced the number of viable *S. mutans* and *S. sanguinis*. Similar results have been observed in other studies. The results of the Comeau et al. study [20] have shown that Log_10_ CFU/mL of *S. mutans* was significantly decreased following Rib-mediated aPDT with blue LED. Moradi et al. [50] reported that aPDT using Rib and curing light was effective in reducing the amount of *Enterococcus faecalis* biofilm. The findings of the Khan et al. study [51] suggested that Rib could be used in aPDT to target various pathogens and microbial biofilm-associated infections, offering a multi-perspective view on its application and challenge. Our findings in the eradication of *S. mutans* planktonic and biofilm cells following treatment with Rib-mediated aPDT are consistent with a recent study that shows both porphyrins and flavins as photosensitizers represent potential target chromophores mediating blue light inhibition of *S. mutans* [52].

Very few studies have investigated the role of postbiotics in regulating oral microbiota. An in vitro study demonstrated showed that Lactobacilli postbiotics can reduce the colonization levels of *Aggregatibacter actinomycetemcomitans*, which is related to periodontitis [53]. On the other hand, the clinical data from the studies conducted by Lin et al. [54, 55] demonstrated that oral lozenges made of viable strains of Lactobacilli such as *L. salivarius* subsp. salicinius AP-32, *L. paracasei* ET-66, and *L. plantarum* LPL28 can increase beneficial microbiota in the oral cavity, reduce the colonization of periodontitis-related bacteria, and increase the levels of salivary immunoglobulin A (IgA). In the present research, we evaluated that LC and LP as the postbiotic mediators can limit the growth rate of oral pathogenic bacteria *S. mutans* at 31.2 µg/mL and 62.5 µg/mL, respectively. More recently, the use of Toluidine blue O (TBO) mediated-aPDT combined with probiotics (*Lactobacillus brevis* and *Lactobacillus plantarum*) has been advocated as another beneficial adjunct to subgingival debridement (SD) in non-surgical treatment and management of periodontitis. The combination of SD + TBO mediated-aPDT + probiotic treatment demonstrated significantly greater reductions in bleeding on probing (BOP), Gingiva-Index simplified (GIs), and red complex bacteria *Porphyromonas gingivalis* and *Tannerella forsythia* compared with patients who received mechanical debridement alone at 6 months [22].

To the best of our knowledge, aPDT in combination with postbiotic mediators (aPDT^+ LP^ and aPDT^+ LC^) has not been employed against oral bacteria. In this study, the antibacterial and inti-biofilm effects of Rib-mediated aPDT plus LC and LP against *S. mutans* planktonic and biofilm cells were evaluated. The results of this study showed that aPDT using LP and LC could reduce the survival of *S. mutans* by 4.9 Log_10_ CFU/mL and 5.2 Log_10_ CFU/mL, respectively. Moreover, it was found that the aPDT^+ LP^ and aPDT^+ LC^ groups demonstrated the highest level of biofilm disruption, followed by CHX. The percentage of reduction observed in aPDT^+ LP^ and aPDT^+ LC^ groups was more than 73%. Our SEM findings were strongly confirmed by the CFU quantitative test results, which assess biofilms. While the obtained data revealed that CHX is so effective, the use of noninvasive, safe, potent, effective, broad-spectrum, and promising alternative modalities to CHX in dentistry including aPDT is necessary due to the limitations and less desirable effects of CHX. The CHX can cause extrinsic staining of teeth, an increase in supragingival calculus formation, allergic reactions, taste changes and tooth staining, tongue irritation and wheezing/shortness of breath, sore mouth and/or throat, and limited use, among others [56]. There have also been concerns about the development of resistance to CHX [57, 58]. Since aPDT targets multiple cellular components within microbial cells, including DNA, lipids, and proteins, no instances of microbial resistance to this treatment approach have been reported so far.

Same as CHX, the conventional aPDT has non-selective killing effects against both pathogenic and commensal (beneficial) microorganisms. This is a limitation of the conventional aPDT, as the elimination of commensal species can disrupt the healthy microbial balance. However, in the modified form of aPDT, such as the DNA-aptamer-nanographene oxide mediated-aPDT [17], this limitation has been addressed. The modified aPDT specifically targets and eliminates pathogenic microbes, while leaving the essential commensal species intact. This selective targeting is a key advantage of the modified aPDT approach compared to the conventional aPDT.

Photoexcited Rib can compromise the redox status of bacterial cells, leading to significant membrane damage and ultimately causing bacterial death. This property of Rib is exploited in aPDT. The mechanism of action involves the generation of reactive ROS when Rib is exposed to light, which induces oxidative damage in cells and tissues [17]. However, the use of postbiotic mediators in photo-induced methodologies can enhance the effectiveness of aPDT by causing structural changes in the cell membrane permeability and leading to cell death.

Our research investigated how aPDT^+ LP^ and aPDT^+ LC^ impact the *gtfB* gene in *S. mutans*, which plays a role in controlling bacterial adhesion to teeth, cell aggregation, coaggregation, and the stability of biofilm [59]. Reducing *gtfB* gene activity might lead to decreased biofilm and plaque formation, ultimately aiding in caries prevention. Our findings show that indicate that photoexcited Rib notably decreased the gene expression of *gtfB*, especially in the aPDT^+ LP^ and aPDT^+ LC^ groups. There were no significant differences in reducing the mRNA expression of *S. mutans* between Rib, LC, LP, and blue light alone compared to the control group. We found that Rib-mediated aPDT with LC and/or LP is equally effective as CHX in reducing virulence gene expression, but it requires a high concentration of 0.2% CHX has limitations due to side effects such as teeth staining, calculus buildup, a metallic aftertaste, and allergic reactions [60]. Therefore, there’s a need for a more biocompatible antimicrobial approach that effectively combats microbial biofilms. Our research shows that aPDT^+ LP^ and aPDT^+ LC^ achieve results similar to CHX without these side effects, making it a promising additional treatment option. While recognizing the constraints of our research, we maintain that this approach could be a useful strategy in targeting various pathogens and microbial biofilm-associated infections.

## Conclusion

It was found that the antimicrobial and anti-biofilm effects of Rib against *S. mutans* can be enhanced by using either more Rib concentration or its activation in low concentration by blue light in aPDT. It can be concluded that the combination of Rib-mediated aPDT with postbiotic mediators (aPDT^+ LP^ and aPDT^+ LC^) decreases the cell viability and disrupts the microbial biofilm *of S. mutans*. Adding these mediators to the Rib mediated-aPDT was accompanied by an additive effect. The aPDT efficacy could be improved by increasing the aPDT dose since there was no evidence of synergism. aPDT^+ LP^ and aPDT^+ LC^ also downregulate the expression of genes involved in the biofilm formation of *S. mutans*, leading to a potential approach for controlling biofilm-associated infections caused by *S. mutans* and ultimately caries. To enhance the robustness of our findings, additional in vitro and in vivo experiments should be conducted to assess the efficacy of aPDT^+ LP^ and aPDT^+ LC^ against different cariogenic bacteria.

## Data Availability

The data of this study is available from the corresponding author on reasonable request.

## Abbreviations

aPDT: Antimicrobial photodynamic therapy
gtf: Glucosyltransferase
LC: Lactobacillus casei
LP: Lactobacillus plantarum
qRT-PCR: Quantitative real-time polymerase chain reaction
Rib: Riboflavin
ROS: Reactive oxygen species
